# Identification of circulating immune landscape in ischemic stroke based on bioinformatics methods

**DOI:** 10.3389/fgene.2022.921582

**Published:** 2022-07-25

**Authors:** Danyang Li, Lifang Li, Fei Quan, Tianfeng Wang, Si Xu, Shuang Li, Kuo Tian, Meng Feng, Ni He, Liting Tian, Biying Chen, Huixue Zhang, Lihua Wang, Jianjian Wang

**Affiliations:** ^1^ Department of Neurology, The Second Affiliated Hospital of Harbin Medical University, Harbin, China; ^2^ College of Bioinformatics Science and Technology, Harbin Medical University, Harbin, China

**Keywords:** biomarker, immune, neuroinflammation, ischemic stroke, machine learning

## Abstract

Ischemic stroke (IS) is a high-incidence disease that seriously threatens human life and health. Neuroinflammation and immune responses are key players in the pathophysiological processes of IS. However, the underlying immune mechanisms are not fully understood. In this study, we attempted to identify several immune biomarkers associated with IS. We first retrospectively collected validated human IS immune-related genes (IS-IRGs) as seed genes. Afterward, potential IS-IRGs were discovered by applying random walk with restart on the PPI network and the permutation test as a screening strategy. Doing so, the validated and potential sets of IS-IRGs were merged together as an IS-IRG catalog. Two microarray profiles were subsequently used to explore the expression patterns of the IS-IRG catalog, and only IS-IRGs that were differentially expressed between IS patients and controls in both profiles were retained for biomarker selection by the Random Forest rankings. *CLEC4D* and *CD163* were finally identified as immune biomarkers of IS, and a classification model was constructed and verified based on the weights of two biomarkers obtained from the Neural Network algorithm. Furthermore, the CIBERSORT algorithm helped us determine the proportions of circulating immune cells. Correlation analyses between IS immune biomarkers and immune cell proportions demonstrated that *CLEC4D* was strongly correlated with the proportion of neutrophils (r = 0.72). These results may provide potential targets for further studies on immuno-neuroprotection therapies against reperfusion injury.

## Introduction

Stroke, which is common among elderly patients, is often associated with a poor prognosis. Ischemic stroke (IS) accounts for up to 87% of the total stroke burden worldwide (Saini et al., 2021), and it represents an increasing economic and health burden as the population ages (Benjamin et al., 2018). To date, clinically approved treatments for acute IS, including intravenous thrombolysis (IVT) and endovascular treatment (EVT), are still limited and restricted with narrow time windows after the appearance of symptoms (Saver and Adeoye, 2021). The main goal of such therapies is to ensure the reperfusion of the ischemic penumbra, a region that remains viable over a limited period before irreversible ischemic neuronal death occurs (Moskowitz et al., 2010). Remarkably, reperfusion has a definite therapeutic effect on IS, and the inclusion of neuroprotection would also revolutionize the treatment of this disease.

IS a disease with a complex and intricate pathophysiology. During the past decades, several trigger elements have been unraveled to be associated with brain injury after IS, including excitotoxic and microvasculature injuries, blood-brain barrier (BBB) disruption, edema, and neuronal death induced by glucose and oxygen deprivation (OGD) (Iadecola et al., 2020; Levard et al., 2021). Besides, immune responses also play an important role in the mechanisms underlying IS. Post-ischemic inflammation can be regarded as the immune system’s response to the disruption of tissue homeostasis (Anrather and Iadecola, 2016). According to the danger theory (Liesz et al., 2015), the release of damage-associated molecular patterns (DAMPs) caused by primary damage to brain cells defines a common pathway that facilitates both innate and adaptive immune responses within the brain and, subsequently, the peripheral circulation (Shi et al., 2019). Over the last 2 decades, researchers have made great progress in characterizing the general pattern of immune responses/inflammation in the brain after IS. Besides, numerous attempts have been made to identify potential biomarkers that may help predict the outcomes or guide the treatment decisions of IS (Xu et al., 2020; Wang et al., 2021; Xu et al., 2021; Li et al., 2022; Zhong et al., 2022). However, there are still several limitations that need to be overcome to obtain a comprehensive understanding of the immune landscape of IS. There have been previous studies on immune responses after IS, most were conducted on experimental models and a few on human post-mortem brain tissue. Thus, the paucity of human *in vivo* data has limited our exploration of this important topic. In addition, there is little clinical evidence of peripheral immune cells mobilization and vascular inflammation (Shi et al., 2019). Given the abovementioned limitations, identifying immune biomarkers of IS patients at the level of the peripheral circulation is a crucial task for unveiling the underlying mechanisms of this complex disease, and may provide molecular grounds for the development of efficient neuroprotective strategies.

In recent years, the principle of “guilt by association” (Oliver, 2000), whose central idea is that functional similar genes and their products often have either physical interactions or functional associations, which plays a prominent role in the development of computer algorithms for predicting novel relative genes based on known disease genes (Wu et al., 2008; Luo et al., 2019; Liang et al., 2021; Zhang et al., 2021; Wang et al., 2022). The STRING database (Keshava et al., 2009) is a reliable database that contains large amounts of molecular protein-protein interaction (PPI) data obtained from various sources. In this network, the associations between proteins are quantified by confidence scores. Thus, based on the associations between potential genes and known IS immune-related genes obtained from the PPI network, it is possible for us to predict more novel genes involved in immune responses after IS.

In this study, we applied the random walk with restart (RWR) algorithm (Köhler et al., 2008), a strategy that belongs to network propagation (Cowen et al., 2017), on the PPI network to identify potential IS immune-related proteins with the input of the previously validated ones we collected. Thus, a gene catalog consisting of both the validated and predicted IS immune-related genes (IS-IRGs) was constructed. Then, differentially expressed genes (DEGs) between IS patients and controls in two microarray profiles were identified for further verification of the IS-IRG catalog we constructed. To screen for key molecular features at the acute phase of IS, we incorporated two machine learning strategies: the Random Forest (RF) algorithm and Neural Networks to the IS-IRGs representing the overlap of DEGs between IS and non-IS samples and the catalog we constructed. Lastly, we established the characteristics of circulating immune cells in each sample using the CIBERSORT algorithm. This study will enhance the understanding of the role of immune responses/inflammation in IS with the ultimate aim of providing a rationale for neuroprotective therapies for IS.

## Materials and methods

### Collection of human validated IS immune-related genes

Using the key search terms “ischemic stroke” AND “immune”, we manually searched the PubMed database (https://pubmed.ncbi.nlm.nih.gov/) for articles published in English at any time before 16 November 2021. Only the *Homo sapiens* species of the immune-related genes was considered. We thoroughly read the 5,980 items that resulted from our searches and picked out the validated human IS immune-related genes that met the following criteria: i) the gene was present in at least three IS samples (including blood and brain tissue samples); ii) the gene was validated through reliable basic biological experimental methods such as RT-PCR and Western Blot; and iii) the gene was differentially expressed at mRNA or protein level (*p* < 0.05). Finally, a total of 76 validated human IS immune-related genes were identified and eventually used as seed genes for identifying novel candidate genes associated with the immunity involved in the pathogenesis of IS.

### PPI network

We downloaded the files named “9606.protein.links.v11.5.txt.gz” and “9606.protein.info.v11.5.txt.gz” from the STRING database (Keshava et al., 2009) to construct the PPI network. The former file contains 11,938,498 human PPI pairs, and each PPI pair includes two proteins displayed by their Ensembl IDs, as well as a confidence score representing the PPI strength. The latter one lists the correspondences between Ensembl IDs and gene symbols. The confidence scores range from 1 to 999. Here, two proteins that are highly functionally associated with each other achieve a high score. Since 900 is the cutoff value for the highest confidence, we only retained PPI pairs with confidence scores of >899. The retained PPIs were used to build the high-confidence PPI network which termed G in subsequent sections.

### Random walk with restart

In the present study, additional genes with the potential for functional association with the seed genes were identified by the algorithm of RWR based on the PPI network.

The mathematical process of the RWR was run through the formula below:

$$P\left(\mathrm{t+}1\right)=\left(\mathrm{1-}\alpha \right)M^{T}P\left(t\right)+\mathrm{\alpha P}\left(0\right)$$

P (0) represents the initial vector, and it contained 19,247 (number of total proteins in PPIs) components. Each component of the vector represented the probability that the corresponding node would be an IS-IRG. Here, P (0), the probability scores of the components that represent seed genes (namely validated IS-IRGs) were set at 1/76 while those of other components were set at 0. M represents the column-wise normalized adjacency matrix of the PPI network, G. The parameter α, a value ranging from 0 to one that represents the probability of the walker returning to the initial nodes, was set at 0.3 according to a previous study (Li et al., 2021). P (t+1) is the network node’s rank in step (t +1) when the probability vector became stable, which was measured using ‖P (t+1) − P(t)‖< 10^−6^, the RWR algorithm stopped and out-put P (t+1) as the result. A gene that was assigned a high probability was more likely to be a potential IS-IRG. We set the threshold 10^–4^ for selecting candidate IS-IRGs.

### Permutation test

Since there are inevitable false positive interactions in the network G, we performed permutation test to authenticate our results. In this test, 1,000 gene sets were randomly produced, each of which comprised 76 Ensembl IDs. Then, 76 Ensembl IDs in each set were used as seed nodes in the RWR algorithm to yield a probability for each node in the network G. After testing all 1,000 gene sets, each gene in network G got 1,000 probabilities. For each candidate IS-IRG g, a measurement called the permutation FDR was calculated using the formula below:

$$\mathrm{FDR}\left(g\right)=\frac{\delta }{\mathrm{1000}}$$

Where δ represents the number of randomly produced sets in which the score of gene g is larger than the score yielded by the validated IS-IRGs. As shown in the formula, candidate genes with high FDR scores are more likely to be false-positive genes because they are not specifically identified by validated IS-IRGs. That is to say, we should select potential IS-IRGs with low *p*-values. Given that 0.05 is the standard threshold *p*-value for statistical significance, we set this value as the threshold FDR value. The remaining genes were designated as potential IS-IRGs.

### Enrichment analysis

Here, we constructed a gene catalog of IS-IRGs by merging validated human IRGs and potential IS-IRGs. To explore the biological functions among IS-IRGs, we applied gene ontology (GO) and the Kyoto encyclopedia of genes and genomes (KEGG) pathway analysis using the R package “clusterProfiler” (version 4.2.0). The GO and KEGG pathway databases originated from the “org.Hs.eg.db” package. The threshold for statistical significance was set at a *p*-value of 0.05 for selecting enriched GO terms and KEGG pathways. The results were visualized using the “ggplot2” R package.

### Datasets collection and data processing

The following two human microarray profiles (GSE16561 and GSE58294) and one high throughput sequencing dataset (GSE102541), which are available in the Gene Expression Omnibus (GEO) database, were used in this study. Details of the selected microarray datasets are presented in Table 1. The GSE16561 and GSE58294 datasets were used to screen for IS immune biomarkers and construct Neural Network models, while the GSE102541 profile was used to verify such a model. Data processing was performed using R (version 4.1.1). The series matrix file of each dataset was downloaded from the GEO database. The R package “AnnoProbe” was used to conduct the quantile normalization and background correction of data. The differentially expressed genes (DEGs) between IS and healthy control samples were identified using the “limma” package with an adjusted *p*-value of <0.05 and a fold-change (FC) of 1.2 as the threshold. The heatmap plots were generated using the “ComplexHeatmap” R package.

**TABLE 1:** Information of GEO datasets.

ID	Sample type	Platform	IS sample	Control sample
Training set				
GSE16561	Peripheral whole blood	GPL6883	39	24
GSE58294	Peripheral whole blood	GPL570	69	23
Test set				
GSE102541	Peripheral whole blood	GPL22755	6	3

### Screening of key immune-related biomarkers of IS

The intersection set of GSE16561 DEGs, GSE58294 DEGs, and IS-IRG catalog was selected as candidate immune biomarkers of IS for further analysis. Random Forest is an ensemble learning algorithm that combines many individual decision trees into a single predictive algorithm. The algorithm repeatedly subsamples the input data to create regression trees that best fit the relationship between predictors and responses. It is a powerful ranking algorithm that was used to identify the key features (immune biomarkers) of IS patients in our study. Using the “randomForest” R package, we calculated the Gini importance scores for candidate immune-related signatures separately in the GSE16561 and GSE58294 datasets according to the respective gene expression values. Subsequently, 25% of all biomarkers with stronger importance in each dataset were retained according to the Gini mean decrease index, while the remaining 75% of them were discarded. By taking the intersection set of biomarkers retained above in the GSE16561 and GSE58294 datasets, IS immune biomarkers were finally identified.

The Neural Network fits a model by taking the predictors as inputs into artificial neurons and firing when the weighted inputs reach a certain level. It is typically used for modeling the complex nonlinear relationship between the dependent and predictor variables. For Neural Network model construction, we transformed the normalized gene expression matrix into a binary gene expression (0,1) matrix. For a sample, if the expression level of an up-regulated IS immune biomarker was equal to or greater than the median expression of this biomarker across all samples, then the matrix value for that biomarker in that sample was assigned as 1, otherwise, it was assigned as 0. A similar pattern, although reversed, was true for down-regulated IS immune biomarkers. The binary matrix was subsequently input into the Neural Network model, which was constructed by the “neuralnet” (v1.44.2) package of R. And the R package ‘NeuralNetTools’ (v1.5.3) was used for visualization. The number of hidden neurons in each layer was set at 5, the activation function was set as “logistic”, and the rest of the arguments were left as default. The classification performance of the combination of immune-related biomarkers in each training set was assessed via the ROC multifactor analysis. The model with the higher AUC was validated in the test dataset. The “pROC” package for R was used to obtain the area under the ROC curve.

### Evaluation of circulating immune cell distribution

The main function of the CIBERSORT algorithm is to infer the infiltrating fractions of 22 sub-types of immune cells from expression profiling referring to leukocyte signature matrix LM22 (Newman et al., 2015). We performed CIBERSORT via the provided R script (https://cibersortx.stanford.edu) using 1,000 permutations without quantile normalization in the local R environment. The v-SVR function was implemented by the “e1071” (version 1.7–9) R package. Only samples with a significant *p*-value (*p* < 0.05) in the CIBERSORT results were considered more accurate evaluations of the immune cell composition, and such samples were picked out for further analysis. All evaluated 22 sub-types of immune cell fractions added up to one for each sample. GSE16561 was used to quantify the infiltrated immune cells at the acute phase of IS.

## Results

### Construction of a human IS-IRG catalog

**FIGURE 1:**
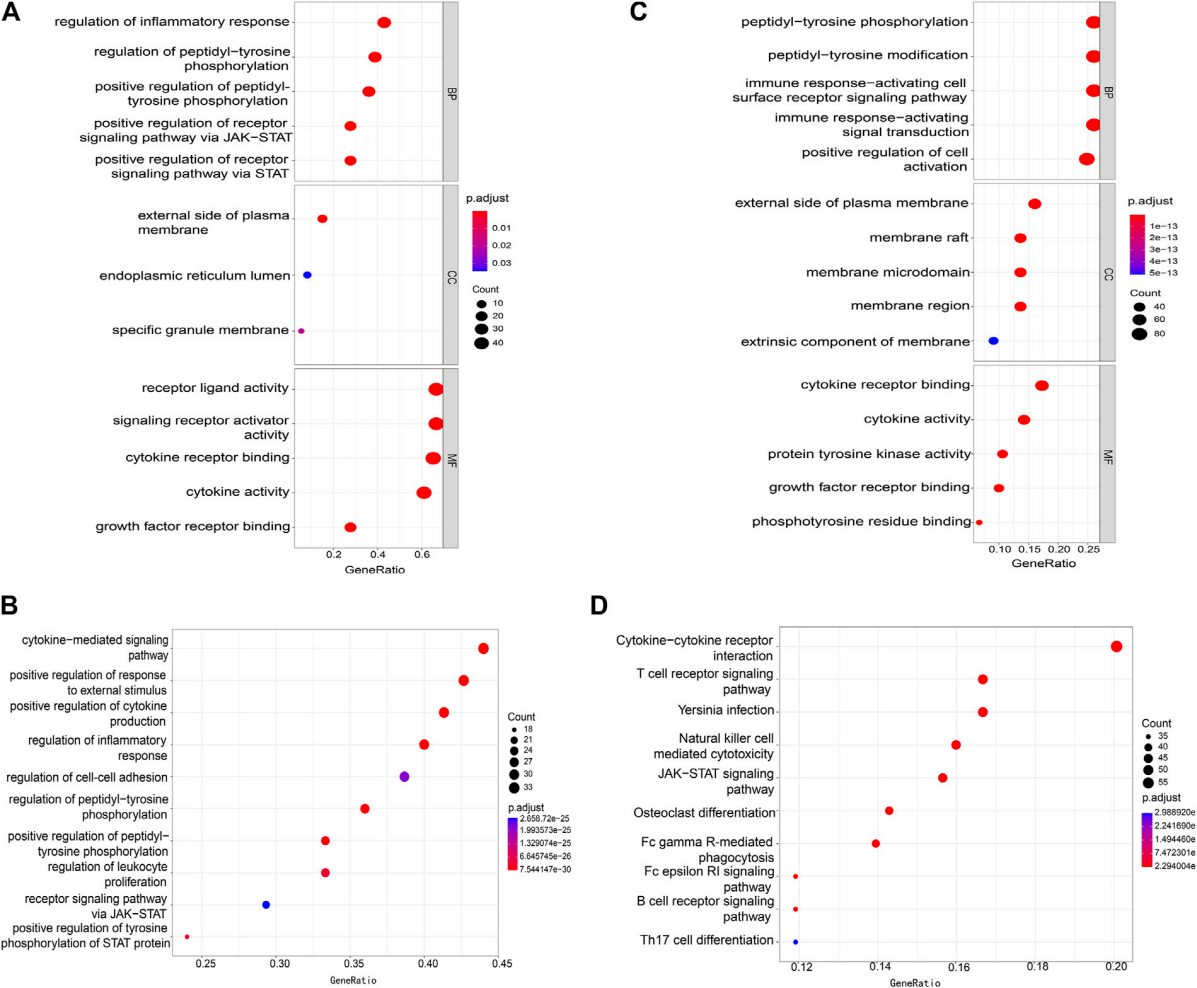
Items of GO and KEGG enrichment analysis. **(A)** GO analysis for validated IS-IRGs. **(B)** KEGG analysis for validated IS-IRGs. **(C)** GO analysis for the IS-IRG catalog. **(D)** KEGG analysis for the IS-IRG catalog. GO, Gene Ontology; BP, biological process; CC, cellular component; MF, molecular function; KEGG, Kyoto Encyclopedia of Genes and Genomes; IS-IRGs, ischemic stroke-immune related genes.

We manually collected a total of 76 genes that were experimentally validated to be associated with the immunopathological process of IS (Supplementary Table S1). Based on these validated IS-IRGs, we performed GO (Figure 1A) and KEGG (Figure 1B) enrichment analyses. The results of the GO enrichment analysis suggested that these validated IS-IRGs were mainly enriched in terms such as “regulation of inflammatory response,” “positive regulation of cytokine production,” peptidyl-tyrosine phosphorylation-associated procedures, and JAK-STAT-related pathways (Figure 1A). We identified a total of 60 KEGG pathways (*p* < 0.05) via the KEGG pathway enrichment analysis. The top ten pathways and the corresponding gene ratios are presented in Figure 1B. KEGG results revealed that the validated IS-IRGs were mainly enriched in the “cytokine-mediated signaling pathway”, “positive regulation of response to external stimulus”, “positive regulation of cytokine production”, “regulation of inflammatory response”, “regulation of cell-cell adhesion”, etc.

To identify more genes that tended to be associated with the immunopathological process of IS, we used the RWR algorithm to the high-confidence PPI network and the permutation test to reduce the number of false positives. In the PPI network G we constructed, 19,247 proteins served as nodes and 247,200 PPIs as edges. Seventy-six validated IS-IRGs were mapped into G as seed genes, after which we used the RWR algorithm in network G to score all genes apart from seed genes. Thus, we generated a ranking list of scores representing the probabilities of each gene in G being a candidate IS-IRG. Genes with probabilities of >10^−4^ were retained, and there were 5,838 such genes. We then used the permutation test to filter out the false-positive genes among the 5,838 genes, which yielded 263 genes (Supplementary Table S2) as the potential IS-IRGs. By merging validated and potential IS-IRGs, a catalog of IS-IRGs was constructed, which contained 339 genes. GO enrichment analysis (Figure 1C) and the KEGG pathway enrichment analysis (Figure 1D) were applied based on the IS-IRG catalog we identified. The GO analysis results of the IS-IRG catalog were similar to those of validated IS-IRGs (Figure 1C). The KEGG analysis revealed that the IS-IRG catalog was enriched in “Cytokine-cytokine receptor interaction”, “T cell receptor signaling”, “*Yersinia* infection”, “Natural killer cell-mediated cytotoxicity” and “JAK−STAT signaling” pathways. (Figure 1D).

### Identification of DEGs and expression patterns of the IS-IRG catalog

**FIGURE 2:**
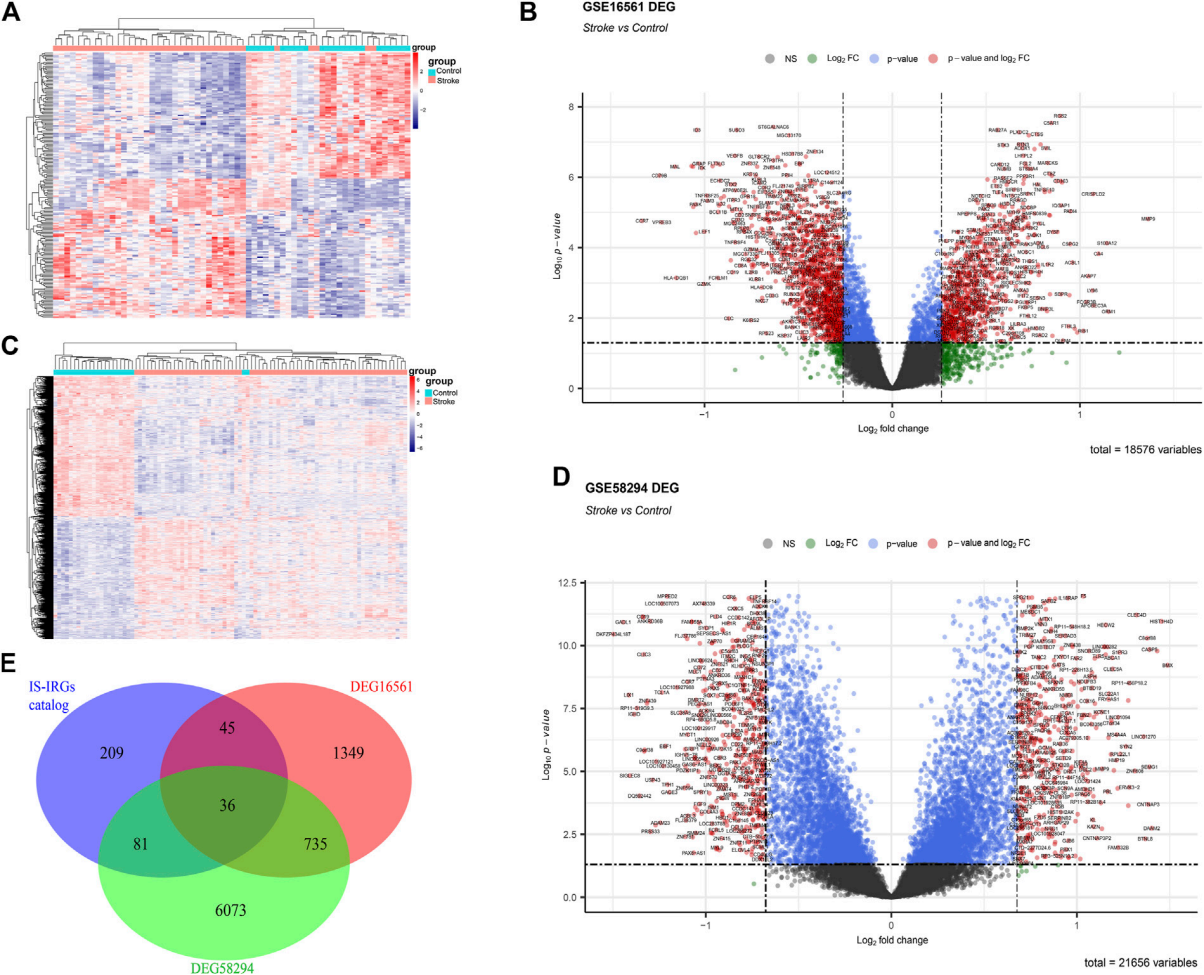
Identification of DEGs and IS immune-related DEGs. **(A,C)** Heatmap showing the differences in DEGs between ischemic stroke patients and controls in the microarray dataset. **(A)** represents the GSE16561 data set and **(C)** represents the GSE58294 data set. **(B,D)** Volcano plot showing the differences in DEGs between ischemic stroke patients and controls in the microarray dataset. **(B)** represents GSE16561 data set and **(D)** represents GSE58294 data set. **(E)** Venn diagram representation of the intersection of the following three sets: DEGs in the GSE16561 dataset, DEGs in the GSE58294 dataset, and IS-IRGs catalog.

The DEGs between IS and healthy control samples in the GSE16561 and GSE58294 datasets were identified using the “limma” package of R with an adjusted *p*-value of <0.05 and FC of 1.2 as the threshold. As shown in Figures 2A, B, we obtained 1,254 down-regulated genes and 911 up-regulated genes from the GSE16561 dataset. In the GSE58294 dataset, a total of 3,510 down-regulated genes and 3,415 up-regulated genes were isolated (Figures 2C, D).

DEGs belonging to the IS-IRG catalog were obtained by the intersection of the following three gene sets: DEGs of GSE16561, DEGs of GSE58294, and the IS-IRG catalog. As a result, we finally obtained 36 genes present in all the three gene sets above, and they were defined as potential IS immune biomarkers for further analyses (Figure 2E). The up-regulated and down-regulated trends of these 36 potential biomarkers followed the same trend in GSE16561 and GSE58294. Among the 36 potential biomarkers, 16 genes were up-regulated and 20 genes were down-regulated, and 10 genes were seed genes while 26 genes were potential IS-IRGs. The details of these biomarkers are shown in Supplementary Table S3.

### Enrichment and correlation analysis of potential IS immune biomarkers

**FIGURE 3:**
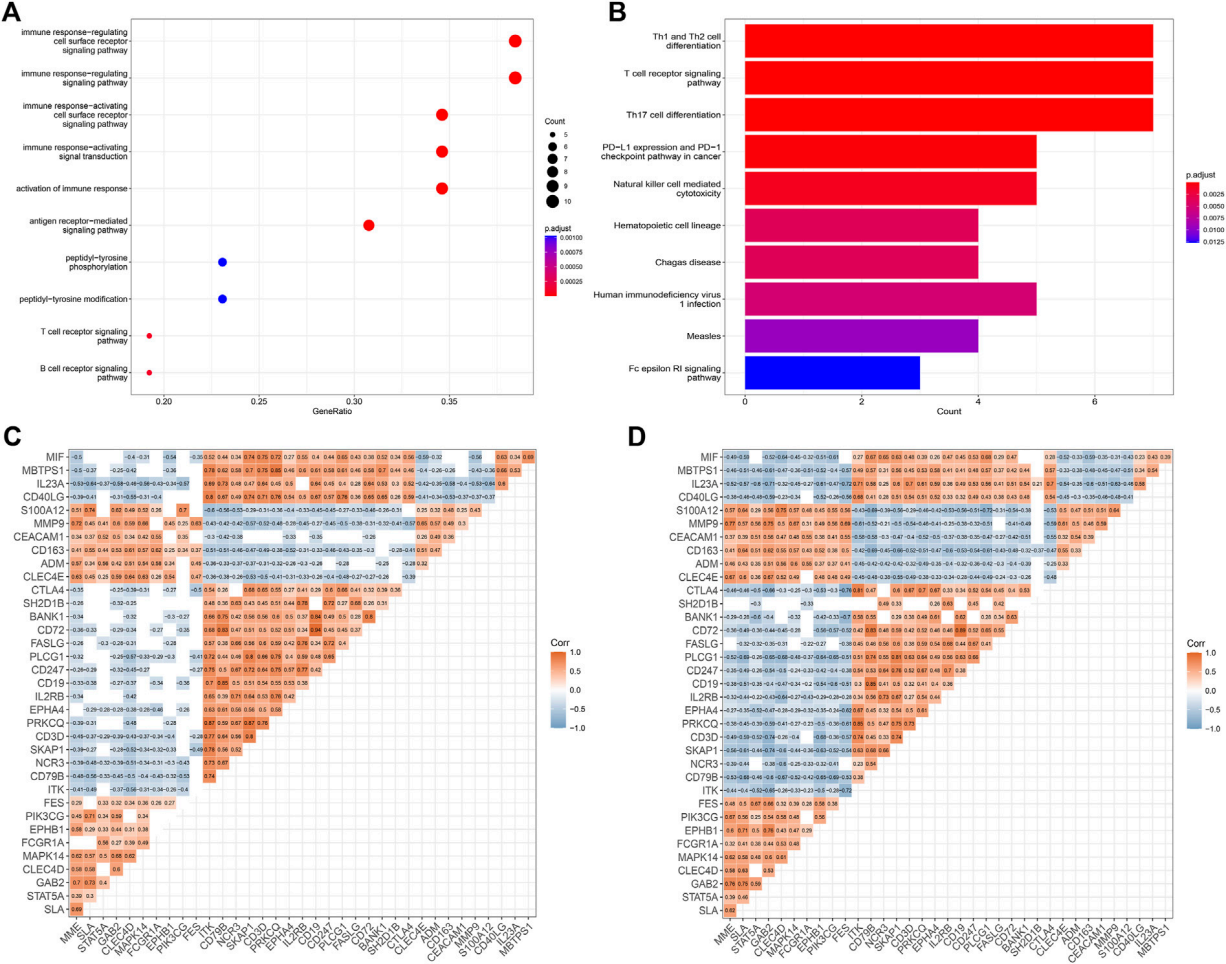
Enrichment and correlation analyses of potential IS immune biomarkers. **(A)** GO analysis of potential IS immune biomarkers. **(B)** KEGG analysis of potential IS immune biomarkers. **(C,D)** Heatmap showing the correlation analysis of potential IS immune biomarkers in the GSE16561 and GSE58294 datasets. The correlation coefficient (Corr) was determined via Pearson’s correlation analysis. Blank spaces indicate that correlations were not statistically significant (*p* > 0.05).

Based on the 36 IS immune-related biomarkers, we performed GO and KEGG enrichment analyses. As shown in Figure 3A, the GO items were mainly enriched in immune response-related procedures, the antigen receptor-mediated signaling pathway, and T cell and B cell receptor signaling pathways. Besides, the top 10 enriched KEGG terms were as follows (Figure 3B): Th1 and Th2 cell differentiation, T cell receptor signaling pathway, Th17 cell differentiation, PD-L1 expression, and PD-1 checkpoint pathway in cancer, Natural killer cell-mediated cytotoxicity, Hematopoietic cell lineage, Chagas disease, Human immunodeficiency virus one infection, Measles, Fc epsilon RI signaling pathway.

A correlation analysis between the potential IS immune biomarkers was performed and the correlations between biomarkers were measured using Pearson’s correlation coefficient. The results showed a similar pattern in the GSE16561 (Figure 3C) and GSE58294 (Figure 3D) datasets.

### Screening of Key Features among IS immune biomarkers

**FIGURE 4:**
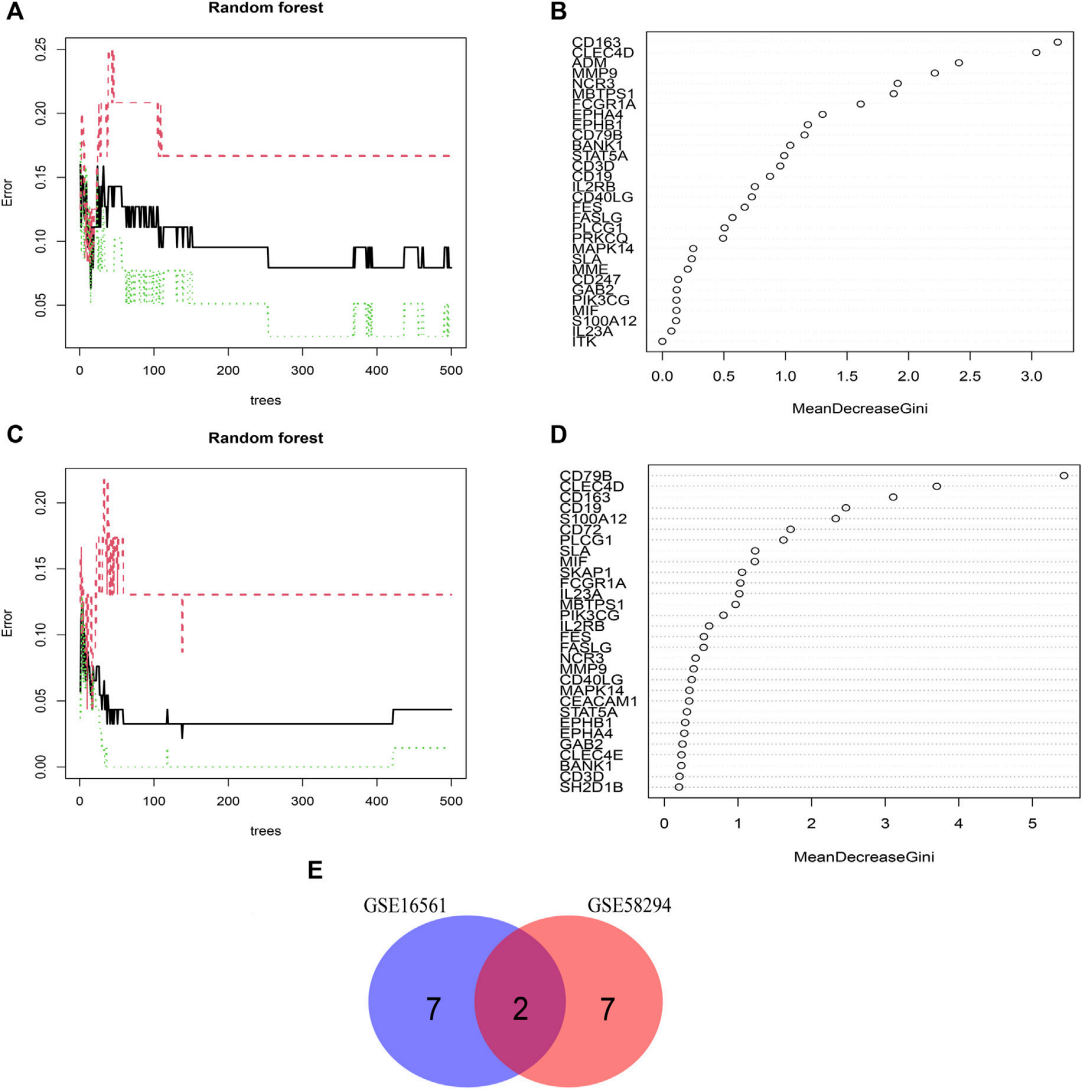
Screening of Key Features of IS immune biomarkers. **(A,B)** Random Forest model screening for key features in the GSE16561 dataset. **(C,D)** Random Forest model screening for key features in the GSE58294 dataset. **(E)** Venn diagram showing the intersection of key features between the GSE16561 and GSE58294 datasets.

We used the RF algorithm to identify the key features of IS immune-related pathogenesis at the level of gene expression. This machine learning method was applied to the sample data of the GSE16561 and GSE58294 datasets. The error was minimum when the number of optionTrees was 15 and 138 in dataset GSE16561 and GSE58294, respectively. According to the Mean Decrease Gini index of each biomarker, the top 25% of all potential IS immune biomarkers with stronger importance in each dataset were retained. The key IS immune biomarkers in GSE16561 were *CD163*, *CLEC4D*, *ADM*, *MMP9*, *NCR3*, *MBTPS1*, *FCGR1A*, *EPHA1*, and *EPHB1* (Figures 4A, B). In GSE58294, the key IS immune biomarkers were *CD79B*, *CLEC4D*, *CD163*, *CD19*, *S100A12*, *CD72*, *PLCG1*, *SLA*, and *MIF* (Figures 4C,D). A total of two IS immune biomarkers were obtained by taking the intersection of these two datasets, which were *CD163* and *CLEC4D* (Figure 4E).

### Classification performance of two key immune biomarkers in IS

**FIGURE 5:**
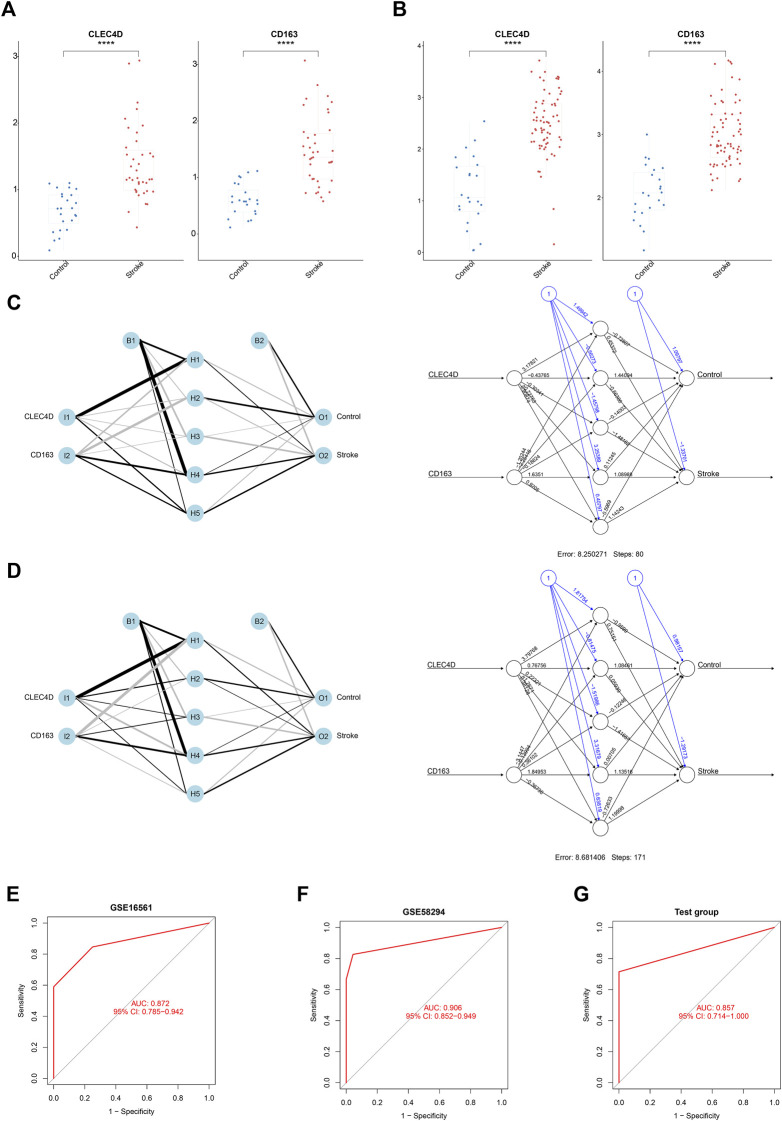
The Diagnostic Value of Three Key Immune-Related Biomarkers in IS. **(A,B)** Box plot analysis showing the expression values of IS immune-related biomarkers in IS patients vs controls. (The *p*-value was obtained from the *t*-test. ****p < 0.0001.) **(C)** Neural Network model constructed using GSE16561. **(D)** Neural Network model constructed using GSE58294. **(E)** ROC curves of the Neural Network diagnostic model constructed using GSE16561. **(F)** ROC curves of the Neural Network diagnostic model constructed using GSE58294. **(G)** Validation of the Neural Network model in the GSE102541 dataset.

Thereafter, we calculated the expression values of these two immune biomarkers in IS patients and normal controls. According to the results, both datasets showed the same trend in the gene expression of *CLEC4D* and *CD163*, which were up-regulated in IS patients compared to normal controls (all *p* < 0.001, Figures 5A, B).

To construct a model with optimal performance in distinguishing between IS and non-IS, we used the Neural Network algorithm to calculate the weight of each immune biomarker we picked. The classification models (which we call “double-biomarkers” here) were constructed separately based on GSE16561 and GSE58294 as we described above (Figures 5C, D). In the hidden layers of the Neural Network model, the weighting factors of each biomarker were calculated and further used in the multifactor ROC analysis. As shown in Figures 5E, F, the “double-biomarkers” had a high accuracy in distinguishing IS patients from healthy controls in GSE16561 (AUC = 0.872, 95%CI: 0.785–0.942) and GSE58294 (AUC = 0.906, 95%CI: 0.852–0.949). The Neural Network model trained on the GSE58294 dataset was then used to the GSE102541 test dataset. Figure 5G shows that this model also had a similar performance in the test dataset (AUC = 0.857, 95%CI: 0.714–1.000).

### Evaluation of the circulating immune cell distribution

**FIGURE 6:**
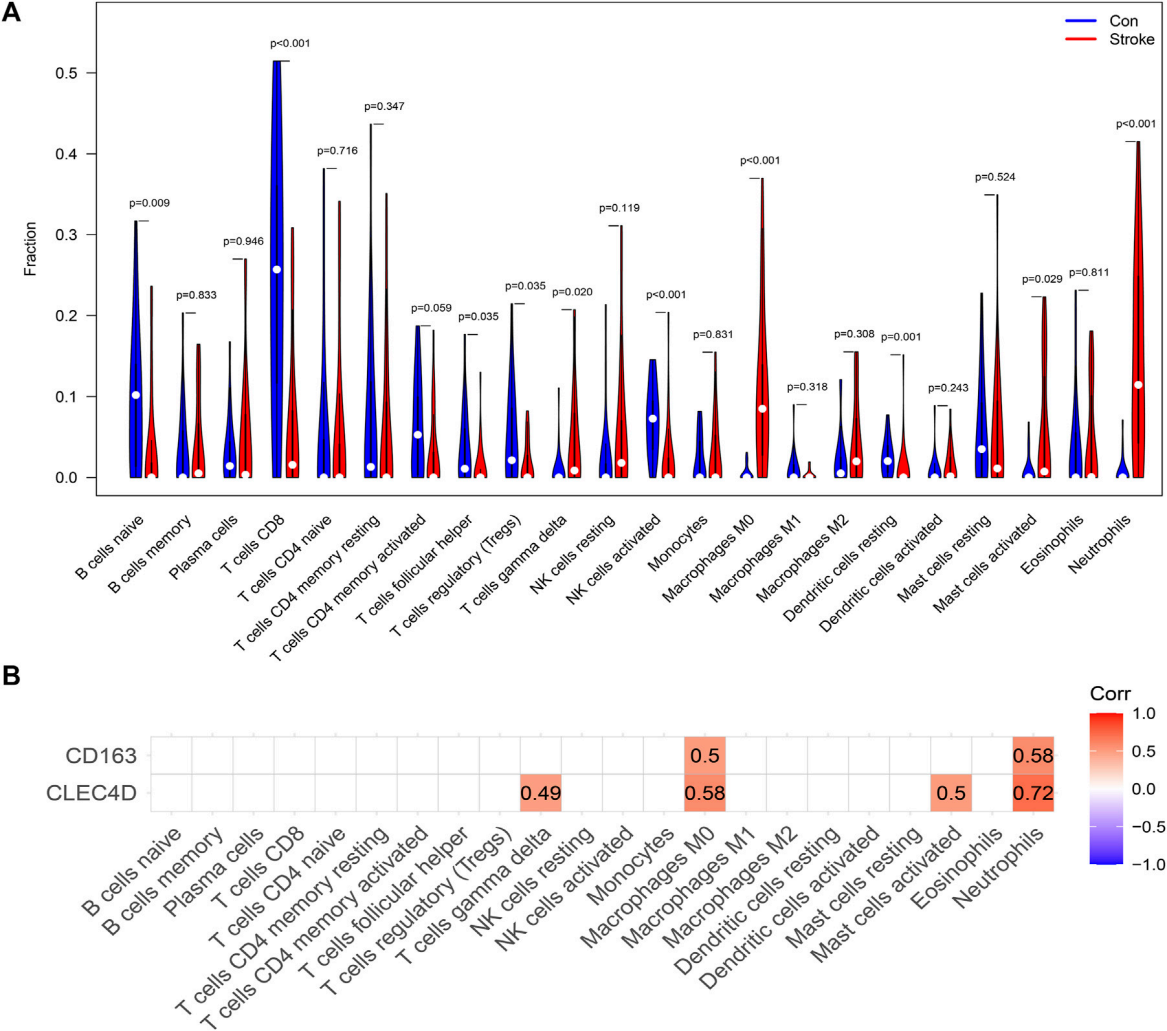
Peripheral Blood Immune Cell Infiltration in IS. **(A)** Violin plot of 22 kinds of immune cells’ differentially infiltrated fractions in peripheral blood between healthy controls and IS patients. **(B)** Heatmap showing the correlation between IS immune-related biomarkers and 22 circulating immune cells. Correlation coefficients were calculated using Spearman’s correlation analysis. The correlation was interpreted primarily according to the magnitude of the correlation coefficient: Corr >0.70 indicates a strong correlation; Corr of 0.50–0.70 indicates a moderate correlation; Corr of 0.30–0.50 indicates a weak-moderate correlation, and Corr <0.30 indicates a weak correlation.

CIBERSORT was used to perform immune subset deconvolution in the GSE16561 dataset. As shown in Figure 6A, the violin plot presents the fractions of each of the 22 kinds of immune cells both in the groups of IS patients and controls. Compared with healthy samples, the IS ones generally contained a higher proportion of macrophages M0 (*p* < 0.001), neutrophils (*p* < 0.001), T cells gamma delta (*p* = 0.020), and activated mast cells (*p* = 0.029), whereas the proportion of naïve resting B cells (*p* = 0.009), T cells CD8 (*p* < 0.001), T cells follicular helper (*p* = 0.035), Tregs (*p* = 0.035), activated NK cells (*p* < 0.001), resting dendritic cells (*p* = 0.001) was relatively lower.

The correlation between biomarkers and immune cells was measured using Spearman’s correlation coefficient, and the results are shown in Figure 6B. The expression of *CD163* demonstrated a moderate positive correlation with the fractions of macrophages M0 and neutrophils. The expression level of *CLEC4D* positively correlated with the fractions of neutrophils, M0 macrophages, activated mast cells, and gamma-delta T cells. Furthermore, it is worth noting that *CLEC4D* was strongly correlated with the proportion of neutrophils (Spearman’s Corr = 0.72).

## Discussion

Inflammation/immune responses to ischemic stroke have become a new hotspot in the field of IS pathogenesis research nowadays. Recent studies have illustrated that systemic leukocytosis is a marker of the inflammatory response after IS (Veltkamp and Gill, 2016). Thus, exploring immune biomarkers and clarifying their correlation with immune cells in peripheral blood may not only be valuable for mechanistic studies, but also suggest novel molecular targets for the treatments of IS. Our group started the present study by collecting previously validated human data and created a catalog of IS immune-related genes incorporating both the validated and predicted data using the RWR algorithm. Then, we reduced our large catalog to a small set of predictive biomarkers with the help of a series of machine learning strategies. Last, the evaluation of the immune cell composition helped us gain insight into the immune landscape of the peripheral blood of IS patients.

Recently, numerous bioinformatic methods variated from network propagation have been proposed for identifying genetic associations. The mathematical propagation processes of these approaches can be summarized as follows: random walk, random walk with restart (RWR) and diffusion kernel (Cowen et al., 2017). The RWR algorithm can transform a short list of seed genes into a genome-wide profile of gene scores based on their proximity to seed genes in a gene network. Furthermore, RWR performs better in capturing the local topology of the interactions in PPI network compared to random walk and diffusion kernel. To study the pathogenesis of different human diseases, several studies applied the RWR on the PPI network to predict novel disease-related genes based on known ones (Zhang et al., 2018; Lu et al., 2019; Liang et al., 2021). In the present study, the RWR and permutation test were applied to achieve a relatively comprehensive inference of novel IS-IRGs. Then, the expression patterns of the IS-IRG catalog were verified using microarray profiles data. It is important to mention here the reason why the GSE16561 and GSE58294 datasets were chosen as verification profiles for the IS-IRG catalog. It is widely known that the critical time points of gene expression profiling are quite necessary for analyzing the pathophysiology of progressive disease. As shown in Supplementary Table S1, the time points of gene expression analysis by biological low-throughput methods are concentrated on the early stage of IS (≤24 h), which is the peak stage of immune response activation, which is why the datasets we mentioned above were chosen for further validation.

We identified a total of 36 IS-IRGs that were differentially expressed in both datasets we mentioned above between IS and control samples. These IS-IRGs were further performed with enrichment analyses, and the results illustrated that the regulation and activation of cell surface receptor signaling pathways were the most enriched GO items. Receptor-ligand interactions have been shown to be widely involved in the immune responses of IS. During the early immune responses of IS, Toll-like receptors recognize components of damaged cells known as DAMPs. Such a receptor-ligand link leads to the activation of the NF-κB pathway and then to the activation of microglia (Cserép et al., 2020). Recent evidence suggests that chemokine and chemokine receptor signaling, such as CC3CL1/CX3CR1 signaling and CCL2/CCR2 signaling, are also present in the processes of peripheral immune cell recruitment into the ischemic brain (Garcia-Bonilla et al., 2016; Wattananit et al., 2016; Cisbani et al., 2018). As for the enriched KEGG pathways, the T cell receptor signaling pathway and Th1, Th2, Th17 cell differentiation were the top items. Several published papers have demonstrated that multiple subtypes of T lymphocytes play a vital role in early inflammation and brain injury following ischemic stroke (Brait et al., 2012). Th1, Th2, and Th17 cells are effector T cells differentiated from naive CD4^+^ T cells (Prass et al., 2003; Liesz et al., 2009). indicated that there is a shift from Th1 to Th2 cytokine production in the peripheral blood of IS patients, and this phenomenon may be due to the “stroke-induced immunodeficiency syndrome” that occurs as early as 12 h after the onset of symptoms and may persist for several weeks. Several previous studies have demonstrated that Th17 cell abundance is elevated in ischemic brain tissue and may indicate a poor prognosis (Dolati et al., 2018; Chen et al., 2021), possibly through the activation of MMPs and BBB breakdown (Arya and Hu, 2018).

To explore a minimum number of IS molecular immune features, we utilized machine learning methods to narrow down the 36 IS-IRGs, which were validated to be expressed differentially between IS patients and controls in microarray datasets, for further detection of key features. The RF algorithm is widely used to address feature ranking problems with an integrated tree classifier kernel. This method can help avoid the problem of overfitting to a great extent and has the advantages of strong model generalizability and excellent accuracy (Albaradei et al., 2021). By constructing RF models through the analysis of two distinct datasets, we identified two IS immune biomarkers, *CLEC4D* and *CD163*. *CLEC4D* (also known as *CLECSF8*, *MCL*) is expressed only in selected populations of myeloid cells (particularly abundant on classical monocytes but relatively rare on dendritic cells and macrophages) and neutrophils within the peritoneal cavity, blood, spleen, and bone marrow (Wong et al., 2011; Graham et al., 2012). As a member of the C-type lectin receptors (CLRs) family, *CLEC4D* has been identified as a pivotal “sensor” on myeloid cells in the host’s defense against fungal and bacterial infections (Kerscher et al., 2016; Wang et al., 2016; Huang et al., 2018; Xue et al., 2019). To date, studies on *CLEC4D* mainly focus on its role in anti-mycobacterial immunity, and it was shown to be required for the induction of *Mincle* following stimulation with TDM (Miyake et al., 2013), through CARD9-dependent NF-κB p65 activation (Zhao et al., 2014). Also, *CLEC4D* can form a heteromeric complex with *Mincle* to regulate anti-mycobacterial immunity (Kerscher et al., 2016); however, the underlying mechanisms still remain unclear. Remarkably, Suzuki et al. found that the expression level of *Mincle* was up-regulated in immune, neuronal, and endothelial cells in human brain tissue after cerebral ischemia (Suzuki et al., 2013). Thus, it might be speculated that *CLEC4D* is involved in the pathogenesis of IS, just like in anti-mycobacterial immunity. Future studies should be aimed at determining the underlying mechanisms. Neural Network is commonly used for binary classification problems in the medical field; thus, we applied this algorithm to discover the diagnostic performance of the combination of the two IS biomarkers and found that the model constructed by GSE58294 performed better than that constructed by GSE16561 in distinguishing IS. Furthermore, the test set GSE102541 using the GSE58294 model also confirmed the distinguishing ability of the Neural Network model.

We also determined the differences in the fractions of circulating immune subsets between IS patients and controls, after which we used Spearman’s correlation analysis to detect the associations between multi-biomarkers and immune cells. Strikingly, *CLEC4D* was strongly correlated with the proportion of neutrophils. Identifying the specific role of *CLEC4D* in the pathogenesis of IS would be an important future research direction.

Additionally, our study still has several limitations. First, our manually collected data set of human IS-IRGs may not be comprehensive as expected. Second, it would be desirable to verify the two IS immune biomarkers through functional experiments *in vivo* and *in vitro*. Nonetheless, we presented a new method to identify several immune biomarkers associated with IS, and these results may provide potential targets for further study of immune neuroprotective therapy against reperfusion injury.

## Data Availability

Publicly available datasets were analyzed in this study. This data can be found here: https://www.ncbi.nlm.nih.gov/geo/GSE16561 GSE58294 GSE102541.
